# The Effect of Different Image Processing Techniques on the Measurement Accuracy of Endodontics File Length

**DOI:** 10.30476/dentjods.2022.95708.1885

**Published:** 2023-09

**Authors:** Sima Nikneshan, Rabieh Boroumand, Nasim Esmaeeli, Ali Azadikhah, Maryam Paknahad

**Affiliations:** 1 Dept. of Oral and Maxillofacial Radiology, School of Dentistry, Shahid Beheshti University of Medical Sciences, Tehran, Iran; 2 Postgraduate Student, Dept. of Oral and Maxillofacial Radiology, School of Dentistry, Shiraz University of Medical Sciences, Shiraz, Iran; 3 Dept. of Periodontics, School of Dentistry, Shiraz University of Medical Sciences, Shiraz, Iran; 4 Dept. of Oral and Dental Disease Research Center, Oral and Maxillofacial Radiology, School of Dentistry, Shiraz University of Medical Sciences, Shiraz, Iran

**Keywords:** Dental digital radiography, Radiographic image enhancement, Endodontics, Root canal

## Abstract

**Statement of the Problem:**

Different software capabilities have been used in digital systems to increase the diagnostic quality of radiographic projections. Considering the availability of different enhancement techniques, it is necessary to determine the suitability of each technique for various diagnostic cases. There is controversy between studies over the effect of different digital enhancement techniques on the accuracy of file length measurements in endodontics.

**Purpose:**

The present *in vitro* study aimed to determine the effect of the software capabilities on the diagnostic accuracy to determine endodontic file lengths in photostimulable phosphor (PSP) radiographs.

**Materials and Method:**

In the present *in vitro* study, standard access cavities were prepared in 44 extracted human single-rooted permanent teeth. An endodontic file was placed in each root canal. PSP sensors were used for digital imaging using the parallel technique. All the images were reviewed on a same monitor; once normally with no software enhancement and once using software manipulations including pseudo-color, sharpness, emboss, and edge enhancement. The distance from the file tip to the rubber stop was measured on the images by an electronic ruler.

**Results:**

Significantly, all of the image enhancement techniques presented shorter measurements comparing to the actual length. The results revealed the significant accuracy of the measured error in the pseudo-color enhancement technique compared to other techniques.

**Conclusion:**

The results revealed significant differences between the initial measurements (the gold standard) and those made on the manipulated radiographs. In all cases, the measurements were significantly lower than the real values. Therefore, none of these digital enhancement techniques can increase the accuracy of file length measurements significantly. However, manipulation with the pseudo-color option resulted in fewer errors compared to other options and the normal images. Hence, for precise measurements of the endodontic file lengths, pseudo-color processing algorithm can be suggested when using PSP sensors.

## Introduction

Radiography is an essential part of the endodontic procedure. Since the final aim of root canal therapy is to completely seal the apex, knowledge about the accurate location of the apex is necessary and can be gained through radiography [ 1 ]. Working length (WL) is defined as ‘‘the distance between a coronal reference point and the point where canal preparation and obturation should end” by American Association of Endodontics. Due to constant changes of WL during procedure, precise measurement of WL is necessary in all the stages of an endodontic treatment [ 2 ]. Determining precise WL during root canal therapy not only results in a proper apical seal, but also facilitates the preparation of the tooth. 

Different techniques are available to determine the WL including radiography, electronic apex locaters and files, the mean tooth length, and paper points [ 3 ]. Application of digital radiography is increasing among dentists [ 4 ]. An important point of digital radiography is the capacity to improve the quality of the images after exposure, using available digital enhancement techniques in digital software programs [ 5 ]. The correct use of these capabilities can improve the image quality [ 6 ]. Considering the availability of different enhancement techniques, it is necessary to determine the suitability of each technique for various diagnostic cases. 

There is controversy between studies over the effect of different digital enhancement techniques on the accuracy of the WL measurements in endodontics. Some previous studies indicated that digital enhancement techniques do not affect the measurement accuracy of the file lengths significantly [ 7 - 10 ]. However, others confirmed that enhancement techniques might increase the accuracy of radiographs in determining the length of the endodontic files [ 11 - 13 ]. Therefore, the present study aimed to evaluate different image enhancement techniques including pseudo-color, sharpness, edge enhancement, and emboss and to determine the best technique for identifying the tooth apex and determining the working length using endodontic files.

## Materials and Method

The present *in vitro* study was performed on 44 human permanent single-rooted teeth extracted for different reasons including periodontal diseases and orthodontic treatments. Single-rooted teeth and straight root canals without any curvature were used into the research. Teeth with root canal calcification and root fractures as well as endodontically treated teeth were excluded. In order to evaluate reliability, the measurements were repeated for 15 samples on both original and manipulated images after two weeks.

### Procedure

**Figure 1:**
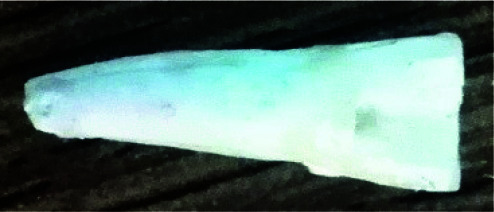
Standard access cavity prepared in a single-rooted teeth

**Figure 2:**
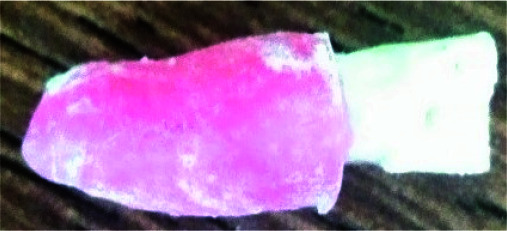
Tooth with a layer of red wax mimicking periodontal ligament (PDL)

**Figure 3:**
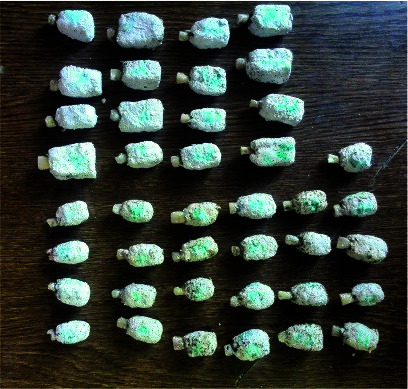
Teeth mounted in a mixture of type I stone and saw dust

**Figure 4:**
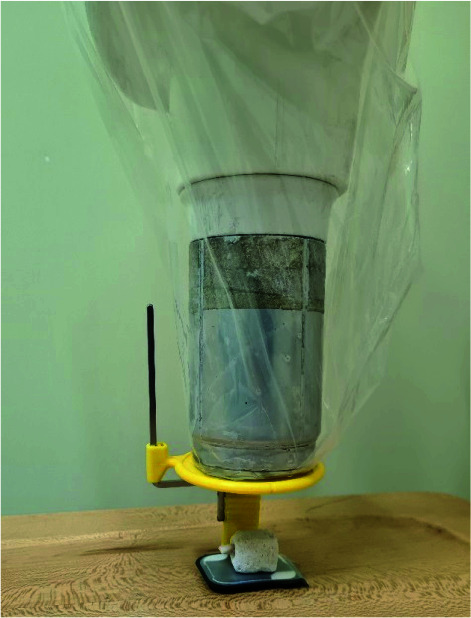
Parallel technique for imaging

Similar to the methods of the previous studies [ 11 - 12 ], standard access cavities were prepared in the 44 single-rooted teeth (Figure 1). An endodontic file was placed into each root canal until the file tip was observed at the apical foramen. The working length from the lower border of the rubber stop to the file tip was recorded by a digital caliper and considered as gold standard of root canal length. A layer of red wax was placed around each tooth in order to simulate the periodontal ligament space and then, the tooth was mounted in a mixture of type I stone and saw dust mixed at a 40:60 ratio (Figures 2-3). A K-file #15 was placed and fixed randomly at lengths ranging 0-2 mm from the root tip in each root canal. For constant imaging conditions, parallel radiographic technique with a single film holder (XCP) was used to fix the tooth and the wireless photostimulable phosphor (PSP) sensor (PSPix Intraoral Digital Imaging System, France). Under identical exposure conditions (kV*p*=65, mA=7, and s=0.100) and an identical position, x-ray irradiation procedures were carried out by using a Prostyle dental x-ray unit (Planmeca OY, Helsinki, Finland). The distance between the sensor and the tooth and between the tooth and the x-ray tube were fixed at 8 and 35 mm, respectively. The x-ray beams were directed by XCP aiming ring at a right angle to the sensor and the teeth which were mounted in a mixture of stone and saw dust (i.e., the parallel technique) (Figure 4). An orthodontic wire measuring 10mm in length was fixed on the mounting surface of the teeth, which was used to calculate and remove the radiographic magnification in order to control the accuracy of each image (calibration factor).

**Figure 5:**
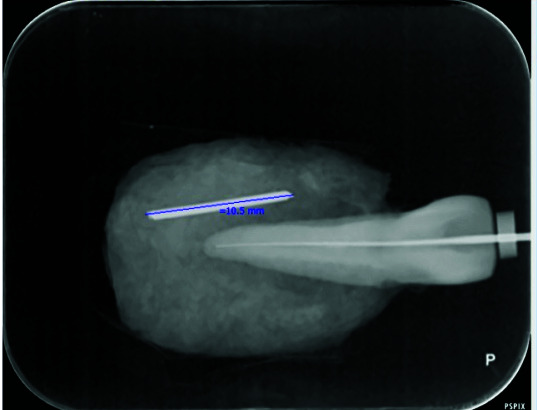
Normal digital radiograph without any software enhancement

**Figure 6:**
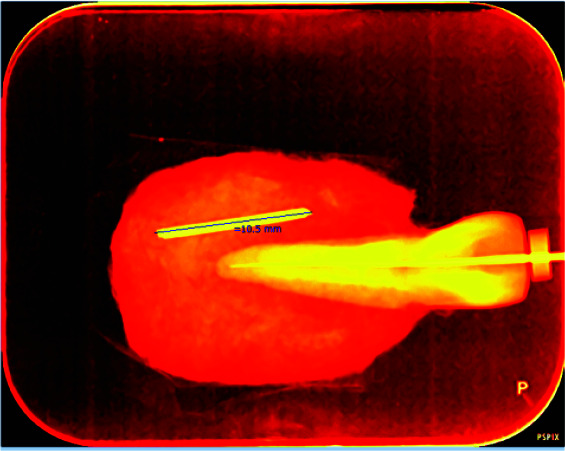
Digital radiograph with pseudo-color enhancement technique

**Figure 7:**
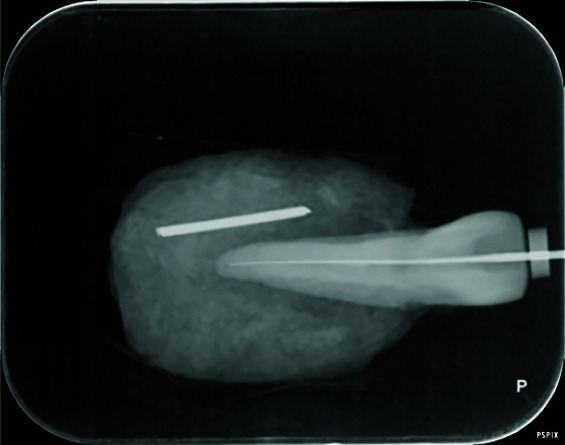
Digital radiograph with sharpness filter

**Figure 8:**
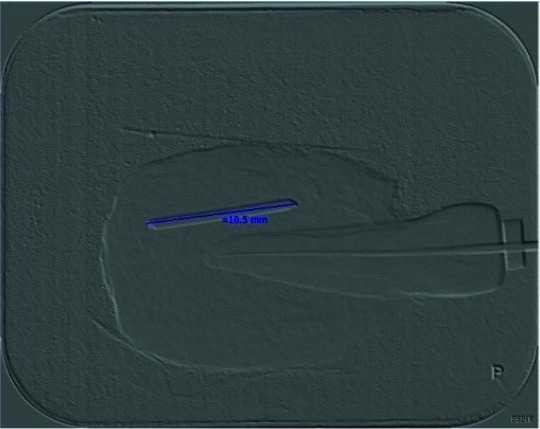
Digital radiograph with emboss filter

**Figure 9:**
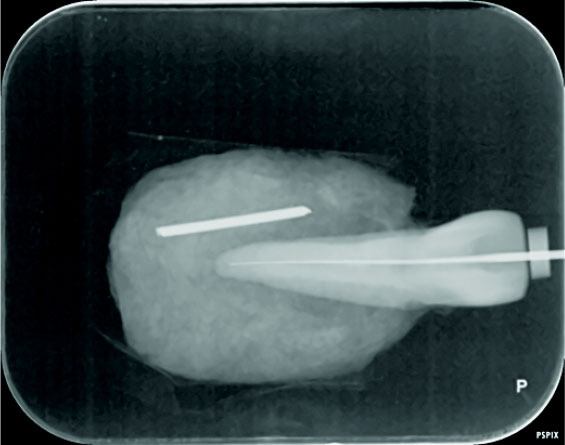
Digital radiograph with edge enhancement technique

The obtained PSP radiographs were scanned by a Digora Optime scanner (Digora Optime, Soredex, Tuusula, Finland). The digital images were viewed under the same environmental conditions using the SCANORA 3.02 software program via a DELL Inspiration 15 laptop (Figure 5) without any software enhancement and with the use of the following software enhancements: pseudo-color, sharpness, emboss, and edge enhancement (Figures 6-9).

The distance between the lower border of the rubber stop and the file tip was measured on the images through an electronic ruler. The measurements were repeated on 15 images by the same observer after two weeks for evaluation of intra-observer reliability. All the images were evaluated by three radiologists and inter- observer reliability was assessed.

### Analysis of data

SPSS (V. 26.0, IBM Corp., Armonk, NY, USA) was used for statistical analyses. A mathematical analysis was used, in which the similarly or reproducibility of each state was assessed based on the gold standard. Repeated-measure of ANOVA was used to evaluate the error of each technique relative to the real length of the file. This analysis showed the manipulation technique that resulted in minimum errors in the measurements. The intra- and inter-observer ICC coefficients were used to assess the reliability of the study.

## Results

ICC coefficient was used to evaluate the similarities among the radiologists. Statistical analyses yielded an ICC coefficient of 0.994 for the initial digital radiographs. The ICC coefficient for emboss, edge enhancement, pseudo-color, and sharpening options were 0.99, 0.993, 0.993 and 0.994, respectively. The ICC coefficients for intra-observer reliability were 0.996 and 0.995 for the initial digital radiographs and those manipulated via the emboss option, respectively. In addition, the ICC coefficients were 0.995, 0.996, and 0.996 for edge enhancement, pseudo-color, and sharpening options, respectively.

Considering the gold standard, the ICC coefficient of the images was 0.985 without manipulation and 0.985 after embossing manipulation. Comparison of the gold standard and pseudo-color, also, yielded a coefficient of 0.985. In addition, comparison of the gold standard and sharpening yielded a coefficient of 0.985. Finally, a coefficient of 0.985 was obtained between the gold standard and edge enhancement. Therefore, these techniques were not significantly different.

Repeated-measure ANOVA was used to compare the measurement errors in different enhancement techniques to the gold standard. The results demonstrated that all the original and manipulated digital radiographs showed significantly shorter lengths than the real values (gold standard). The descriptive statistics of the data have been presented in Table 1. Additionally, the errors in the normal state without manipulation and other radiographs with manipulation have been shown in Table 2. Based on the results, the most frequent measurement errors occurred at 1‒1.5 mm intervals in the normal state and also in employing the emboss, edge enhancement, and sharpening filters. However, for pseudo-color filter, the most frequent errors occurred at 0.5‒1 mm interval, with an error range of 0.1‒1.95 mm.

**Table 1:** Error values(mm) of different enhancement techniques for measurement of endodontic file length in comparison to gold standard using repeated measure ANOVA index

	N	Minimum	Maximum	Mean	Std. deviation
Diff. normal	44	-2.05	-.10	-1.0011	.41798
Diff. emboss	44	-2.15	-.10	-.9784	.41377
Diff. edge	44	-1.95	-.10	-.9807	.40336
Diff. pseudo	44	-1.95	-.10	-.9102	.41702
Diff. sharp	44	-1.95	-.10	-.9739	.40527
Valid N (list-wise)	44				

**Table 2:** The number of samples in each measurement error interval for different digital radiographs comparing to gold standard

Type of digital radiograph filters	The number of samples in each measurement error interval				Total
	1.5 mm<	1.0-1.5 mm	0.5-1.0 mm	0-0.5 mm	
Normal without filter	6(13.6%)	13(29.6%)	21(47.7%)	4(9.1%)	44
Emboss	5(11.4%)	13(29.5%)	22(50.0%)	4(9.1%)	44
Edge enhancement	5(11.4%)	13(29.5%)	22(50.0%)	4(9.1%)	44
Pseudo-color	7(15.9%)	18(40.9%)	15(34.1%)	4(9.1%)	44
Sharpening	6(13.6%)	13(29.5%)	21(47.8%)	4(9.1%)	44

Based on the results described above, repeated-measures ANOVA showed significant differences between the original digital radiographs and those manipulated with pseudo-color and sharpening options. In addition, a significant difference was observed between the edge enhancement and pseudo-color options as well as between emboss and pseudo-color options. Furthermore, a significant difference was found between the pseudo-color and other options. Finally, the images manipulated via sharpening were significantly different from the original radiographs and those manipulated through the pseudo-color option.

## Discussion

The precise measurement of the working length can affect the final verification of the results in endodontic procedure, and consequently, improve the potential of successful treatment [ 14 ]. On a radiograph, digital measurements are considered more accurate than measurements made with an endodontic ruler, increasing the chances of a successful endodontic procedure. Comparative studies are crucial to determine which diagnostic tools are most effective, since the dentists have a wide variety of tools at their disposal [ 9 ]. Each diagnostic task requires optimal parameters for image enhancement, and the use of these parameters affects image quality differently [ 13 ]. This makes it possible to compare original and manipulated images and reduce possible changes resulted from pre-processing [ 7 ]. The present study evaluated the effects of manipulation of digital PSP radiographs through software options including sharpening, pseudo-color, edge enhancement, and emboss on the accuracy of endodontic file length measurements. The results revealed significant differences between the initial measurements (the gold standard) and those made on the manipulated radiographs. In all cases, the measurements were significantly lower than the real values. Kal *et al*. [ 11 ] evaluated the effects of changes applied by different digital enhancement algorithms on the measurement accuracy of endodontic file lengths. Comparing to the actual length of each file size in their study, significantly shorter measurements were obtained by the application of all algorithms for image enhancement, which was in agreement with the findings of the present study. This might be explained by the similarity of the receptors, type of used teeth, applied radiographic techniques. Similarly Shooshtari *et al*. [ 12 ] concluded that sharpening filters did not affect the accuracy of file length in endodontic procedures and could not considered as preferred filters for measurement of file length. Farhadi *et al*. showed that high-level sharpness-processing filter significantly influence the accuracy of endodontic file length measurements [ 14 ]. Mehdizadeh *et al*. [ 8 ] and Oliveira *et al*. [ 9 ] reported significantly different measurements between enhanced digital images and the actual value. However, Woolhiser *et al*. [ 10 ] showed no significant differences in the file measurement accuracy of enhanced and unenhanced digital images. In addition, the results of ANOVA showed the least measurement errors on the radiographs after manipulation with the pseudo-color option. 

Generally, the factors that can affect the identification of file tips on radiographs include the file size, image sensor, exposure duration, and secondary radiation, differences in density, root canal shape, and bony trabeculae [ 15 ]. The results of the present study demonstrated that all the digital radiographs showed shorter lengths than the real values. Therefore, such manipulations on digital radiographs decreased the accuracy of endodontic file measurements. Considering the emboss option, this can be explained by the fact that inverting images results in a decrease in image details, decreasing the observer’s perception of the image. In other words, superimposition of bony trabeculae that are radiopaque can compromise the identification of the file tip in the apical third of the root canal. This becomes more difficult by applying image inversion manipulation. Such factors may result in errors in identifying the file tip, because observers’ eyes are not familiar with the gray appearance of endodontic files and bony trabeculae. 

The present study showed that the enhanced images underestimated the file lengths, which is in line with the results of Kal *et al*. study [ 11 ]. Some previous studies demonstrated that the enhanced images tend to overestimate the endodontic file length, in contrast with the results of the present study [ 8 - 9 , 12 , 14 ]. The difference could be explained due to using different processing software, different types of image receptor, teeth preparation, tooth mounting procedures, and different endodontic files. 

Some previous studies also investigated the effects of popular filters including contrast, emboss, and sharpness. However, these filters were assessed separately and only few studies have compared different filters. In the current research, the pseudo-color filter that has clinically attracted the attention of some endodontists was evaluated and compared with other filters. New findings were obtained in this study, which were related to the applicability of this filter compared to other filters in determining the length of #15 files, as the most standard and commonly used files for determining the working length. Based on the results, the pseudo-color filter exhibited minimum errors among the other filters including sharpness, edge enhancement, and emboss. 

The measurement accuracy of size 08 and 10 files was not significantly increased by any image enhancement techniques applied in Kal *et al*.’s study [ 11 ]. It has been stated that size 08 and 10 files are not suitable for working length measurements in radiography, since the tips of the small files fade away and are usually not visible [ 11 ]. Therefore, the number 8 and 10 endodontic files were not used in the present study.

The *in vitro* nature of the study does not allow the complete generalization of the results to clinical situations. Changes in soft tissues were not simulated on the radiographs in the present study due to the absence of soft tissues that could alter the visual characteristics of the digital radiograph. Not all the software capabilities in the digital radiography systems were evaluated. The other limitation was reliance on subjective assessment of images. Therefore, further studies are required to be conducted on other software programs for measuring endodontic file lengths with the use of different digital systems under different conditions and characteristics of the software program. Furthermore, clinical studies with the use of teeth with curved root canals, which is closer to clinical situations and use of endodontic files with smaller sizes and different alloy structures is recommended.

## Conclusion

The results indicated that the enhanced images underestimated the file lengths compared to the real length. However, manipulation via the pseudo-color filter resulted in fewer errors compared to other filters.

## Conflict of Interest

The authors declare that they have no conflict of interest.
